# Metagenomic analysis of the gut microbiota in *Cygnus cygnus* and isolation, identification, and safety assessment of *Bacillus*

**DOI:** 10.3389/fmicb.2026.1898323

**Published:** 2026-07-16

**Authors:** Ying Xiao, Yabin Lu, Yue Hu, Rui Shi, Duo Mai, Ruixue Lv, Juan Pan, Yuhan Pan, Jiacheng Tan, Zhihui Hao, Jinquan Wang

**Affiliations:** 1 College of Veterinary Medicine, Xinjiang Agricultural University, Ürümqi, China; 2 Xinjiang Key Laboratory of New Drug Research and Development for Herbivorous Animals, Ürümqi, China; 3 Xinjiang Key Laboratory of Animal Infectious Diseases, Ürümqi, China

**Keywords:** *Bacillus subtilis*, *Cygnus cygnus*, metagenomic sequencing, probiotics, safety evaluation

## Abstract

**Introduction:**

As a national second-class protected wild animal, the intestinal microbial community of *Cygnus cygnus* is highly important for health status and ecological balance. The potential application value of probiotics in animal health and disease prevention has attracted much attention, but few studies have investigated probiotics derived from wild animals.

**Methods:**

We initially collected fecal samples before and after the migration of the *C. cygnus* for macrogenomic sequencing. We subsequently isolated *Bacillus* spp. from *C. cygnus* feces and determined their hemolytic properties and tolerance to acid and bile salts to identify potential candidates. We subsequently studied the position of a candidate in phylogenetic trees using 16S rRNA sequences, as well as its susceptibility to antibiotics, toxicity, and effects on animal health.

**Results:**

Metagenomic analysis revealed that the abundance of the *Firmicutes* phylum tended to decrease after the migration of *C. cygnus*, whereas the relative abundance of the *Fusobacteria* phylum increased. Although the diversity and abundance of the gut microbiota of *C. cygnus* remained relatively balanced before and after migration, the microbial community structure changed significantly after migration. These changes were related to reductions in carbohydrate metabolism and energy metabolism, as well as a decrease in the abundance of genes encoding glycoside hydrolases. Twelve strains were isolated and screened, and two strains, *Bacillus subtilis* S07a and N1B, without hemolytic activity were found to have good tolerance to acid and bile salts. It was sensitive to 14 kinds of antibiotics, but *B. subtilis* S07a inhibited on three common pathogenic bacteria. Animal studies have shown that *B. subtilis* S07a (1 × 10^9^ CFU/mL) is safe for use in mice. It also has anti-inflammatory potential and enhances intestinal barrier function to meet the probiotic and safety requirements of probiotics.

**Conclusion:**

Metagenomic analysis revealed reduced abundance of carbohydrate-degrading microbes in the gut of *C. cygnus* post migration. The isolated *B. subtilis* S07a exhibits desirable *in vitro* probiotic potential and satisfactory *in vivo* safety.

## 1 Introduction

China is an important wintering and breeding ground for wild *Cygnus cygnus* and warty-nosed *C. cygnus*. Xinjiang is located in the western migration area of migratory birds in China, and is an important node connecting the migration route of Eurasian migratory birds. *Cygnus cygnus* is a globally endangered species, that belongs to China’s National Grade II protected species (Wang et al., 2021). *Cygnus cygnus* is a large migratory waterfowl whose migration consumes a large amount of energy, causes certain wear and tear on the body, and is exposed to different environments and pathogens, leading to changes in the gut microbiota and immune system (Li et al., 2021).

The gut microbiota is a complex ecosystem that participates in various forms of physiological activities and plays an important role in maintaining the health of the host. The gut microbiota in wild animals not only affects the metabolism, immune regulation, and disease defense of the host but also serves as a natural reservoir of human pathogens (Levin et al., 2021). Moreover, owing to the transregional migratory behaviors of *C. cygnus*, these organisms are likely to become vectors of infection for many diseases, such as the spread of bacteria or pathogens, which allow them to reach different environments and intermediate organisms (Palinauskas et al., 2022). The high geographical dispersal ability and wide distribution of birds make them integral indicators in ecosystems. Stringent environmental selection pressures, high energy expenditure, and long-distance migratory processes endow birds with a unique gut microbiota with complex immune functions (Zhao et al., 2022). The gut microbiota of wild animals is diverse and rich (Cho and Lee, 2020). Deng et al. (2023) studied the gut microbiota of giant pandas and reported that the abundance of *Firmicutes* and *Proteobacteria* is high and that and these microorganisms play a role in the decomposition of cellulose. The gut microbiota composition in wild animals is shaped by multiple factors including host species, diet, habitat, migration stage, and exposure to different environments (Levin et al., 2021). The study of species-specific microorganisms helps to protect wild animals in a targeted manner (Huang et al., 2022). Studies on *swan geese*, a long-distance migratory waterfowl closely related to the *whooper swan*, have shown that its gut microbiota encodes proteases and detoxification enzymes involved in the metabolism of bacterial toxins and environmental pollutants. This adaptation highlights the close link between host species phylogeny, ecological strategy, and the functional composition of the gut microbiota (Wu et al., 2018). Thus, the study of the gut microbiota is helpful for understanding the adaptation and evolution mechanism of *C. cygnus* to changes in the ecological environment. At present, few studies have investigated the gut microbiota of *C. cygnus*, and the community structure and functional changes in the gut microbiota before and after the migration of *C. cygnus* are not yet clear.

Probiotics are defined as live microorganisms that can confer a health benefit to the host (Hill et al., 2014). It has been broadly applied to improve host health in human health care, agriculture, and aquaculture for decades (Hoyt et al., 2019; Fragoso ados Santos et al., 2015). More recently, probiotics have been considered for use with endangered wildlife organisms (Voolstra et al., 2021; McKenzie et al., 2018). *Bacillus* can not only produce antibacterially active ingredients, inhibit the colonization of pathogenic bacteria in the intestine (Warnecke et al., 2012), and maintain the intestinal microecological balance, but also secrete a variety of hydrolytic enzymes, improve the environment in the digestive tract, improve the feed utilization rate, improve the intestinal immunity of animals, and enhance the disease resistance of animals (Wu et al., 2023). *Bacillus* helps maintain the close connection between intestinal epithelial cells by enhancing the expression and function of closely linked proteins, thus preventing the leakage of harmful substances and maintaining the integrity of the intestinal barrier (Wang et al., 2021). Certain *Bacillus* strains, such as those of *Bacillus subtilis* and *Bacillus licheniformis*, have been granted generally recognized as safe (GRAS) status by the U.S. Food and Drug Administration for specific uses (Shleeva et al., 2023). However, the role of *Bacillus* in promoting animal health and the intestinal microbiota remains to be determined.

Therefore, on the basis of metagenomic sequencing technology, we explored in depth the gut microbiota of *C. cygnus* before and after migration, aiming to elucidate its diversity and structural characteristics. *B. subtilis* was isolated and identified from the feces samples of *C. cygnus*, its biological characteristics were screened out, and strains with beneficial potential, and its safety was comprehensively evaluated to determine whether strain S07a can be developed and applied as a wildlife-derived probiotic supplement. This study also aimed to screen novel probiotic strains with excellent properties that can serve as probiotic resources for human health and livestock breeding.

## 2 Materials and methods

### 2.1 Sample collection

All samples in this study were obtained during regional surveillance of key wildlife infectious diseases in Xinjiang (Table 1). Fecal samples collected from *C. cygnus* were screened for highly pathogenic avian influenza virus; only virus-negative specimens were subjected to downstream analyses.

**Table 1:** Sampling information.

Sampling position	Sample grouping	Sampling time	Sampling numbers	Migration time
Yili *Cygnus cygnus* Spring Wetland Park	M1	March 2023	6 (M01-06)	Before the migration (prior to spring departure)
Manas National Wetland park	M2	December 2023	6 (N01-06)	After the migration (upon arrival at wintering habitats)
Yili *Cygnus cygnus* Spring Wetland Park	Y1	March 2023	6 (Y01-06)	Before the migration (prior to spring departure)
Yili *Cygnus cygnus* Spring Wetland Park	Y2	December 2023	6 (E01-06)	After the migration (upon arrival at wintering habitats)

For brevity, Yili *Cygnus cygnus* Spring Wetland Park is abbreviated as the “Y site,” and Manas National Wetland Park as the “M site” in the following text.

### 2.2 Metagenomic data analysis

The total DNA of the fecal samples was extracted using a TIANamp Stool DNA Kit with a fecal DNA extraction kit (centrifugal column type). The genomic DNA sample and a DNA fragment of approximately 350 bp were obtained by a Covaris ultrasonic crusher. Subsequently, the library was prepared, including DNA terminal repair, A-tail, joint sequencing, and PCR amplification. The Qubit 2.0 fluorescence quantifier was used for initial screening of the concentration, and the library was diluted to a standardized working concentration of 2 ng/μL. The insertion fragment distribution was detected through an Agilent 2,100 biological analyser to confirm that the fragment length met the quality standard of 350 ± 50 bp. Used fluorescence quantitative PCR technology for accurate quantification (the effective concentration of the library was >3 nM) to ensure that the library met the PE150 sequencing requirements.

The raw sequencing data obtained on the NovaSeq platform are processed by the fastp tool to generate clean data that can be used for subsequent analysis. MEGAHIT is used to serially assemble the clean data after quality control. After the scaffold was generated, it was further cut at the N-base connection site, and continuous sequential fragments (scaftigs) without N bases were obtained. MetaGeneMark was used to predict the open reading box, redundancy was removed with CD-HIT, gene abundance was calculated by Bowtie2 comparison, and finally, core gene analysis, correlation between samples and Wayne chart analysis were carried out on the basis of abundance information. MetaGenomeSeq and LEfSe analyses were used to identify intergroup differences in species, MetaGenomeSeq was used to hierarchically test for intergroup differences (*p*/*Q* values), and LEfSe analysis was performed with LEfSe software; finally, random forests were used to screen species according to the abundance gradient to construct a model, the importance was evaluated through mean decrease accuracy/min, after which the values were cross-adjusted (default 10-fold), and receiver operating characteristic (ROC) curve was constructed to evaluate the predictive effectiveness.

### 2.3 Isolation and morphological identification of probiotics

Fresh fecal samples of *C. cygnus* were collected from the Manas National Wetland Park in Xinjiang. Three fecal samples were mixed, and 2.5 g of the mixture was suspended in 20 mL of sterilized saline (0.85% NaCl), followed by heating at 80 °C for 20 min to select for spore-forming *Bacillus* spp. After centrifugation (4 °C, 3000 rpm, 3 min), 200 μL of the supernatant was mixed with 800 μL of LB broth and 800 μL of MRS broth (Hopebio, China) and then incubated at 37 °C for 24 h. Serial dilutions were prepared and plated onto LB agar and MRS agar plates (Hopebio, China), followed by incubation at 37 °C for 24 h. Single colonies were picked and subcultured 2–5 times to obtain pure cultures. After Gram staining, the morphology of bacteria isolated from individual colonies was evaluated under a microscope.

### 2.4 Hemolysis test

Sterile defibrous sheep blood was used to prepare sheep blood tablets for later use. In the flat plate line method, the strain was inoculated on blood agar culture medium and cultured at 37 °C for 36 h to observe whether a hemolytic ring was present. The nonhemolytic strain was further verified through the use of Columbia blood agar culture medium to verify whether hemolytic activity was present.

### 2.5 Acid and bile salt tolerance

To determine the acid resistance of the strain, 1 mL of bacterial solution was removed and the supernatant was discard. A PBS solution with a pH of 2.0, 3.0 or 5.0 was added, and the cells were cultured for 2 h. In the same way, PBS solution with 0.1, 0.2 and 0.3% bile salt concentrations was added, and the cells were cultured for 2 h. Growth was monitored by determining the OD_600nm_ in a spectrophotometer (Synergy HTX BioTek, USA).

### 2.6 Antibiotic susceptibility and antibacterial test

Bacterial susceptibility to antibiotics was determined using the Kirby-Bauer disk diffusion method (Bauer et al., 1966). Briefly, bacteria were grown in LB broth at 37 °C for 10 h and then spread onto LB agar plates. Antibiotic disks (Hangzhou Binhe Microorganism Reagent, China) containing standard concentrations of penicillin G, ampicillin, imipenem, amikacin, gentamicin, kanamycin, ciprofloxacin, levofloxacin, tetracycline, clindamycin, chloramphenicol, vancomycin, erythromycin, compound, sulfamethoxazole, and rifampin were placed onto the plates. All the LB plates were incubated at 37 °C for 24 h, after which the diameters of the inhibition zones were measured.

Sterile cotton swabs were used to dip the suspension of *Staphylococcus aureus*, *Escherichia coli* K99 and rat typhoid *Salmonella* evenly on the surface of the LB solid culture medium, after which the sterile Oxford cup was placed on the culture medium and added to the cow cup. Two hundred microliters of fresh isolated strain suspension was cultured at 37 °C for 24 h (the *B. subtilis* S07a suspension was adjusted to 1 × 10^8^ CFU/mL). Each isolated bacterial strain was tested 3 times. The diameter of the bacteriostatic circle was measured, and the antibacterial activity of the strain was determined according to the diameter of the bacteriostatic circle (Suwannaphan, 2021).

### 2.7 Growth rate

The fresh isolated strain solution was inoculated into LB broth medium at 2%, which was subsequently cultured at 37 °C and 180 rpm, after which the absorbance at OD_600nm_ was detected every 2 h, after which the culture was continuously measured for 24 h to determine the growth characteristics of the isolated strain.

### 2.8 Biochemical and molecular biological identification of *Bacillus*

With the use of a *Bacillus* biochemical identification kit, the experimental steps were carried out according to the instructions of the kit, and the results were compared with those of the “Berger’s Bacterial Identification Manual.” Molecular biological identification was performed using 16S rRNA sequence analysis. High-quality genomic DNA was extracted from bacterial isolates using the EasyPure Bacteria Genomic DNA Kit (TransGen Biotech, China). Afterward, the 16S rRNA gene was partially amplified by polymerase chain reaction (PCR) using the universal bacterial primers 27F (5′-AGAGTTTGATCCTGGCTCAG-3′) and 1492R (5′-GGTTACCTTGTTACGACTT-3′). The PCR products were subsequently sent to Bioengineering (Shanghai) Co., Ltd. for 16S rRNA sequencing. A sequence similarity search was conducted using GenBank BLAST. The phylogenetic tree was constructed using MEGA7 software with bootstrap analysis using 1,000 replications to assess the relative stability of the branches.

### 2.9 Animal ethics and experimental design

Three- to four-week-old Kunming mice, male and female, weighing 24 ± 2 g, were purchased from the Experimental Animal Center of Xinjiang Medical University (animal qualification number: SCXK2018-0002). The animal experiment plan was approved by the Ethics Committee of Xinjiang Agricultural University, and the approval number is 2024012.

### 2.10 Acute toxicity

After they adapted to the environment for 7 d, 28 Kunming mice were randomly divided into 2 groups, with 14 in each group (7 males and females). The 1st d control group was irrigated with physiological saline (0.2 mL/d), and the experimental group was supplemented with 1 × 10^9^ CFU/mL *B. subtilis* S07a (0.2 mL/d); the mice were observed for 7 d, after which they were allowed to eat and drink freely. The weight changes, hair color, overall mental state, secretions and excrement status of the mice were recorded every day after gastric lavage. After the 7th day of death, anticoagulant-treated blood was collected for routine blood cell analysis. Each organ was collected to calculate the organ index, which was calculated as follows: organ index (%) = 100 × organ mass (g)/body mass (g). The liver, spleen and kidney were selected for paraffin sectioning and HE staining.

### 2.11 Subacute toxicity

After 56 Kunming mice had adapted to the environment for 7 d, they were randomly divided into 4 groups, with 14 in each group (7 males and females); the control group was irrigated with physiological saline (0.2 mL/one), the low-dose group was treated with 1 × 10^7^ CFU/mL *B. subtilis*, the medium-dose group was treated with 1 × 10^8^ CFU/mL *B. subtilis*, and the high-dose group was treated with 1 × 10^9^ CFU/mL *B. subtilis* S07a (0.2 mL/piece), which was continuously infused for 28 d, and the mice were allowed to eat and drink freely. During the experiment, the weight changes, hair color, overall mental state, secretions and excrement status of the mice were recorded every day after gastric lavage. After the 28th day of death, anticoagulant-treated blood was collected for routine blood cell analysis. Each organ was collected to calculate the organ index, which was calculated as follows: organ index (%) = 100 × organ mass (g)/body mass (g). The liver, spleen, kidney and duodenum were selected for paraffin sectioning and HE staining.

### 2.12 Quantitative reverse transcription-polymerase chain reaction

Total RNA was extracted from colon tissues using TriQuick according to the manufacturer’s instructions. Next, the extracted RNA was reverse transcribed into cDNA, which was amplified and detected by SYBR Green real-time polymerase chain reaction using a Fast 7500 instrument. The relative expression of each target gene was calculated using the 2^−△△Ct^ method with GAPDH as the internal reference.

### 2.13 Statistical analyses

All the data were statistically analysed by one-way analysis of variance (ANOVA) between multiple groups using SPSS 26.0 and GraphPad Prism 8.0.2. The data are expressed as the mean ± standard deviation (mean ± SD) and were considered to be statistically significant at *p* < 0.05.

## 3 Results

### 3.1 Sequencing results

DNA was successfully extracted and sequenced from 24 *C. cygnus* feces samples, and the sequencing data are shown in Supplementary Table S1. The filtered data were assembled and analysed, and the results were counted (Table 2).

**Table 2:** Statistics of the assembly results.

Sample ID	Total len (bp)	N50 len (bp)	N90 len (bp)	Max len (bp)
Y01	233,592,310	1,145	563	788,761
Y02	46,047,797	6,772	715	262,976
Y03	212,821,726	2,092	595	365,513
Y04	232,694,003	825	542	569,312
Y05	365,701,298	761	539	255,571
Y06	211,517,542	1,495	560	378,133
E01	390,222,133	784	539	208,565
E02	248,973,932	750	532	494,801
E03	421,135,764	748	538	494,801
E04	483,791,392	1,022	561	585,900
E05	492,428,781	822	549	304,808
E06	558,351,276	805	538	432,896
M01	108,359,972	2,624	648	251,926
M02	468,624,729	1,831	602	493,304
M03	116,453,676	1,332	563	244,663
M04	100,718,121	935	536	455,522
M05	98,974,099	2,619	575	249,185
M06	59,354,297	2,163	591	221,179
N01	520,359,339	765	540	82,904
N02	102,026,232	1,255	575	803,543
N03	116,576,274	1,158	545	415,216
N04	116,092,694	1,163	545	248,099
N05	89,666,506	825	541	283,114
N06	77,277,831	852	544	247,894

### 3.2 Effects of migration on the microbial diversity of *Cygnus cygnus*

**Figure 1:**
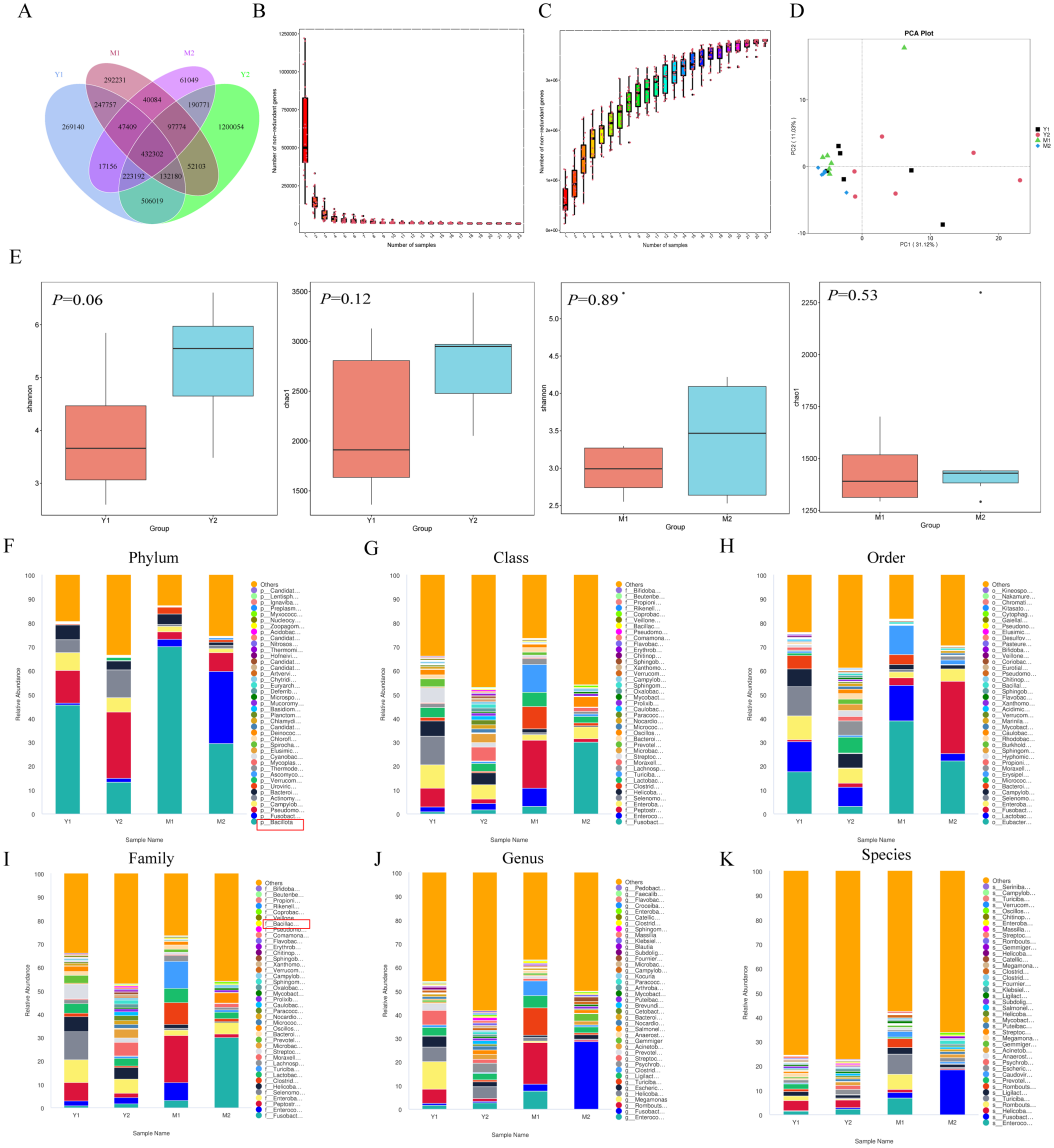
Relative abundance of intestinal microbial species before and after the migration of *Cygnus cygnus* (*n* = 6). **(A)** Venn diagram of non-redundant genes across the four groups. **(B)** Core gene accumulation curve. **(C)** Pan-gene accumulation curve. **(D)** PCA results based on the Micro_NR level. **(E)** Alpha diversity analysis statistical results. Microbial composition and identification of taxa before and after the migration of *Cygnus cygnus* (*n* = 6). Proportion of the abundance of the gut microbiota in *Cygnus cygnus* at the phylum level **(F)**, class level **(G)**, order level **(H)**, family level **(I)**, genus level **(J)**, species level **(K)**.

The results of metagenomic sequencing revealed differences in the abundance of the gut microbiota before and after the migration of the *C. cygnus*. More than 78% of the intestinal microorganisms of the *C. cygnus* are bacteria, and the rest are viruses, archaea, fungi and other classifications. The Venn diagram analysis of non-redundant genes across the four groups revealed that Y2 harbored the largest number of unique genes, reflecting its highest genetic specificity. All four groups shared 432,302 core genes, and the gene overlap between Y1 and M1 was notably higher than that of other pairwise comparisons (Figure 1A). The core gene accumulation curve revealed that the number of core non-redundant genes decreased gradually and plateaued as the number of samples increased, indicating that the core gene set of the microbiome was sufficiently covered by the current sample size (Figure 1B). The pan-gene accumulation curve rose continuously with increasing sample size and tended to stabilize at the later stage, suggesting that the present samples captured most of the gene diversity of the studied microbiome (Figure 1C). PCoA revealed that there was a significant difference in the community structure of the Y1 and Y2 groups. The community structure of group M was quite different from that of group Y, and there are also certain differences between groups M1 and M2 (Figure 1D). To better understand the diversity of species in each group, the alpha diversity before and after the migration of the *C. cygnus* was analysed according to the Shannon and Chao 1 indices. There was no significant difference between the Shannon index and the Chao 1 index of the groups before and after migration (Figure 1E), indicating that the diversity and abundance of the gut microbiota of the *C. cygnus* before and after migration were relatively balanced.

To further analyse the impact of migration on the gut microbiota of *C. cygnus*, this study analysed the gut microbiota at six different classification levels. At the phylum level, the dominant phyla of the Y1 group were *Bacillota*, *Pseudomonadota* and *Campylobacterota*; the dominant phyla of the Y2 group were *Pseudomonadota*, *Bacillota* and *Actinomycetota*; the dominant phyla of the M1 group were *Bacillota*, *Bacteroidota* and *Pseudomonadota*; and those of the M2 group were *Fusobacteriota*, *Bacillota* and *Pseudomonadota*. At the class level, *Clostridia*, *Bacilli* and *Gammaproteobacteria* were more abundant in the Y1 group; *Gammaproteobacteria* and *Actinomycetes* were more abundant in the Y2 group; *Clostridia* and *Bacilli* were more abundant in the M1group; and *Fusobacteria* and *Clostridia* were relatively abundant in the M2 group. At the order level, the Y1 group had higher abundances of *Eubacteriales*, *Lactobacillales* and *Campylobacterales*; the Y2 group had higher abundances of *Lactobacillales*, *Enterobacterales* and *Micrococcales*; the M1 group has a higher abundance of *Eubacteriales*, *Lactobacillales* and *Erysipelotrichales*, and the M2 group had higher abundances of *Fusobacteriales* and *Enterobacterales*. At the family level, the abundance of *Selenomonadaceae*, *Enterobacteriaceae* and *Peptostreptococcaceae*is high in the Y1 group, whereas *Enterobacteriaceae*, *Helicobacteraceae* and *Lactobacillaceae* were abundant in the Y2 group. The abundance of *Clostridiacea*e in the M1 group was greater. The M2 group includes *Fusobacteriaceae*, *Enterobacteriaceae* and *Lactobacillaceae*, among which *Lactobacillus* and *Bacillus*, which are mostly probiotics, are annotated before and after migration. At the genus level, the dominant bacteria in the Y1 group included *Megamonas*, *Romboutsia* and *Helicobacter*; the dominant bacteria in the Y2 group included *Helicobacter*, *Psychrobacter*, *Ligilactobacillus*, *Turicibacter* and *Enterococcus*; and the abundance of *Fusobacterium* in the M1 group was relatively high. At the species level, the strains with the greatest abundance in the Y1 group were *Helicobacter brantae*, the abundance of *H. brantae* in the Y2 group was reduced, and the abundance of the M1 group was *Enterococcus cecorum* and *Turicibacter* sp. TJ11, *Romboutsia timonensis*, and the M2 group had the greatest abundance of *Fusobacterium mortiferum* (Figures 1F–K).

**Figure 2:**
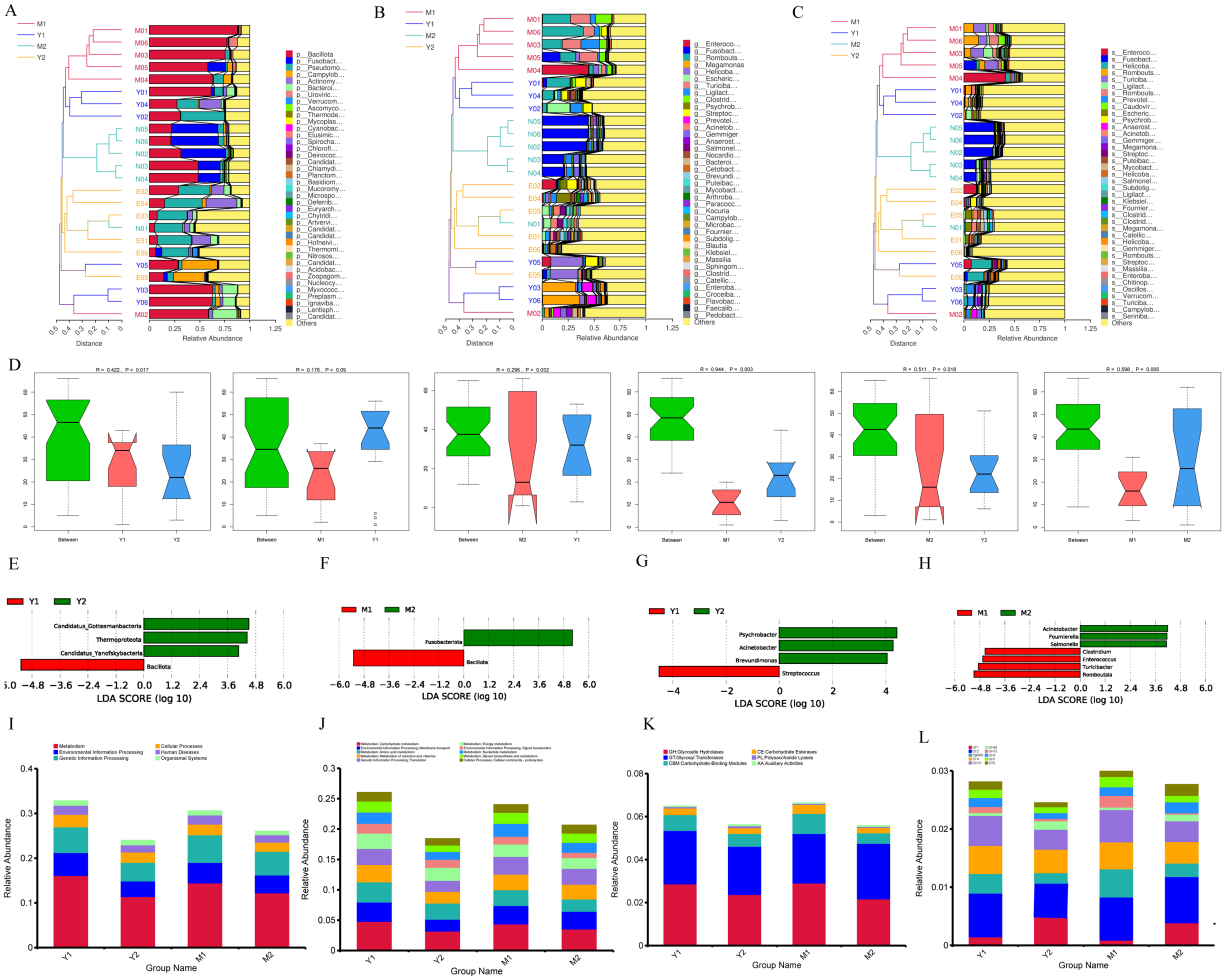
Identification of key microbial taxa before and after the migration of *Cygnus cygnus* (*n* = 6). Difference analysis of the abundances of the gut microbiota before and after the migration of *Cygnus cygnus* at the phylum level **(A)**, genus level **(B)**, and species level **(C)**. Anosim analysis based on species annotation level **(D)**. Distribution map of the LDA values of the significantly different species. LDA value distribution map of different species before and after migration in the Y area at the phylum level **(E)** and genus level **(F)**. LDA value distribution map of different species before and after migration in the M area at the phylum level **(G)** genus level **(H)**. KEGG functional annotation relative abundance chart level 1 **(I)** and chart level 2 **(J)**. CAZy functional annotation relative abundance chart level 1 **(K)** and chart level 2 **(L)**.

On the basis of the abundance of genes in each sample, the Bray-Curtis distance matrix was used to display the relative abundance integration of the cluster analysis between samples at the phylum, species and genus levels of the species. The samples of the Y1 and M1 groups were relatively clustered in the tree diagram at the three levels, indicating that the community structure of the large *C. cygnus* before migration was similar, and Y2 and M2 also showed a certain degree of aggregation, indicating that the community structure of the large *C. cygnus* after migration was similar. At the phylum level, the abundance of the *Firmicutes* phylum in the Y2 and M2 groups after the migration of the *C. cygnus* was significantly lower than that in the Y1 and M1 groups before the migration of the *C. cygnus*, while the relative abundance of the genus *Clostridium* increased after the migration of the *C. cygnus*. At the genus level, the genera *Enterococcus* and *Clostridium* were predominant in the Y1 and M1 groups before the migration of *C. cygnus*, and their relative abundance was relatively high. After migration, the relative abundance of the genus Y2 and M2 group *Clostridium* increased significantly, becoming the main classification unit, while the proportion of *Enterococcus* was relatively reduced, and the distribution of other classification units was also slightly different. At the species level, the abundance of caecal *Enterococcus* in the Y2 and M2 groups after the migration of the *C. cygnus* was significantly lower than that of the Y1 and M1 groups before the migration of the *C. cygnus*, while the abundance of Clostridium death increased significantly (Figures 2A–C). ANOSIM analysis at the species taxonomic level was performed to quantify intra- and inter-group microbial variation (Figure 2D). The ANOSIM *R*-value ranges from −1 to 1; an *R*-value > 0 indicates greater dissimilarity between groups than within groups, and *p* < 0.05 denotes statistically significant inter-group differentiation. All pairwise comparisons yielded positive *R*-values. The comparison between M1 and Y1 presented *p* = 0.09 (non-significant), while all other pairwise group comparisons revealed *p* < 0.05. Collectively, these results confirmed lower intra-group community variation within the same migratory stage, which statistically explained the tight clustering of M1/Y1 (pre-migration) and M2/Y2 (post-migration) samples observed in the hierarchical clustering tree, verifying the validity of our grouping design.

To investigate the specific microbial taxa groups of *C. cygnus* before and after migration, LEfSe analysis was conducted to identify differential microbial biomarkers between groups. LEfSe analysis of the LDA values revealed that the *Firmicutes* phylum of the Y1 and M1 groups was significantly enriched before migration and that the Y2 groups *Candidatus_Gottesmanbacteria*, *Thermoproteota*, and *Candidatus_Yanofskybacteria* after migration of Skybacteria were significantly enriched, and the M2 group *Clostridium phylar* was significantly enriched (LDA score>4). Before migration, Y1 group *Streptococcus* was enriched. After migration, *Psychrobacter*, *Acinetobacter*, and *Brevunmdimonas* were significantly enriched in the Y2 group. Before migration, the abundances of the M1 groups *Clostridium*, *Enterococcus*, *Turicibacter*, and *Romboutsia* were significantly enriched, and the abundances of *Acinetobacter*, *Foumierella*, and *Salmonella* were significantly enriched in the M2 group after migration (LDA score>4) (Figures 2E–H).

The functions of the enriched KEGG pathways included metabolism, environmental information processing, genetic information processing, cellular processes, and human diseases and others. Compared with those of the Y1 group, the metabolic function of the Y2 group still dominated, but the abundance and genetic information, environmental processing and other functions were lower than those of the Y1 group; compared with those of the M1 group, the metabolism, genetic information processing and environmental information processing functions of the M2 group were lower. Further refine the functional categories. Compared with those of the Y1 group, the carbohydrate metabolism and energy metabolism of the Y2 group were relatively weak. Compared with those in the M1 group, the relative abundance of genes related to carbohydrate metabolism and energy metabolism in the M2 group was lower (Figures 2I,J). The distribution of the relative abundance of carbohydrate active enzymes in *C. cygnus* revealed that the relative abundance of glycoside hydrolases in the Y1 and M1 groups was relatively high. The abundance of GT2, GT4 and GH13 in the Y1 group was greater than that in the Y2 group, and the abundance of GT1 in the Y2 group was greater than that in the Y1 group; M1 was enriched with GT2, CBM50, GH13 and GT4, and the abundance of GT2, CMB50, GT4 and GH13 was greater in the M2 group (Figures 2K,L).

**Figure 3:**
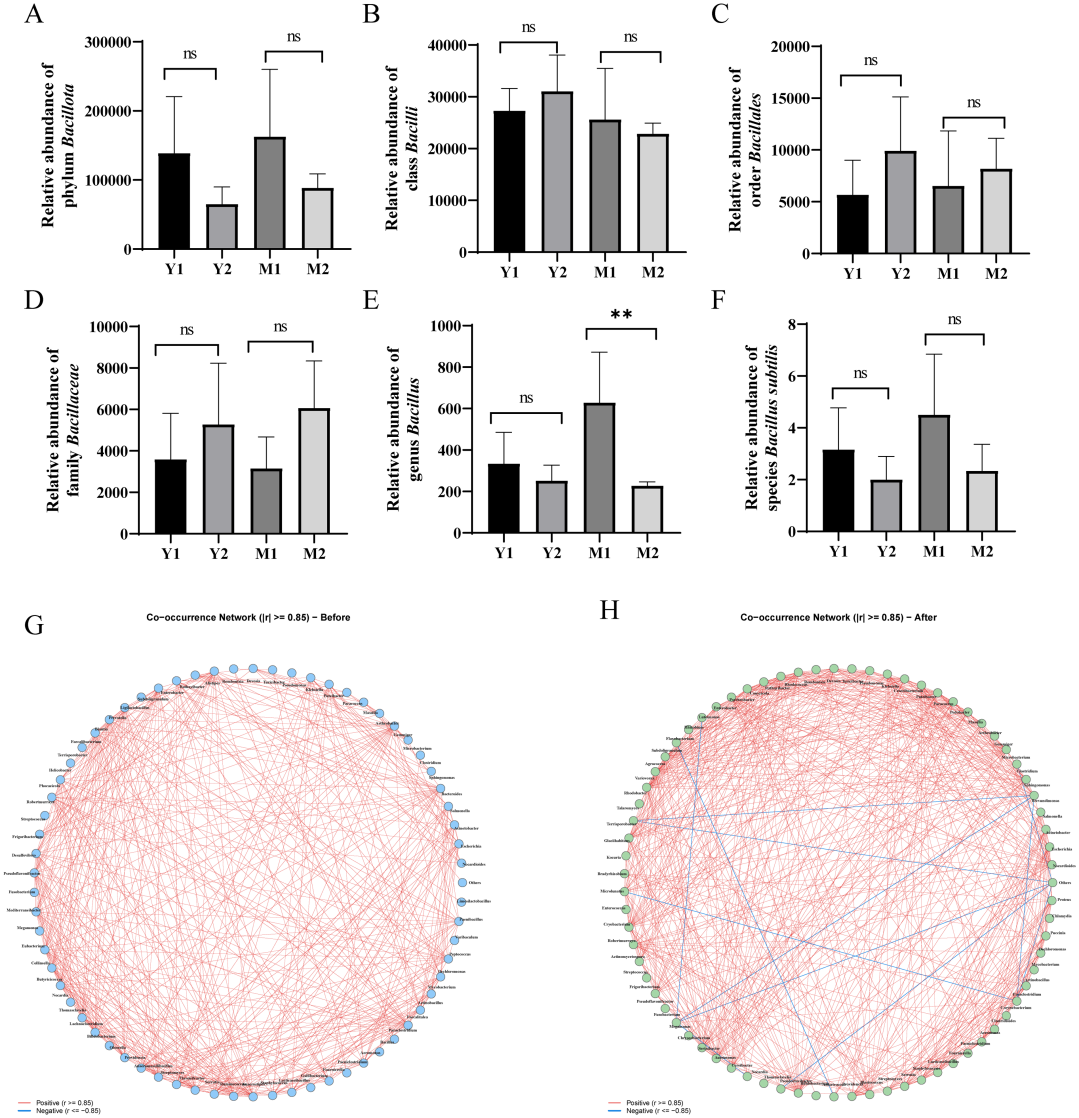
Relative abundance of taxa within the *Bacillota* lineage across six successive taxonomic ranks. **(A)** Phylum *Bacillota*; **(B)** Class *Bacilli*; **(C)** Order *Bacillales*; **(D)** Family *Bacillaceae*; **(E)** Genus *Bacillus*; **(F)** Species *Bacillus subtilis*. ns: *p* > 0.05, **p < 0.01. **(G)** Co-occurrence network of gut microbiota before migration; light blue circles represent individual bacterial genera. **(H)** Co-occurrence network of gut microbiota after migration; light green circles represent individual bacterial genera.

We quantified the relative abundance of the complete taxonomic lineage within *Bacillota*, spanning phylum, class, order, family, genus and species ranks. No significant pairwise differences in relative abundance were detected at the phylum *Bacillota* (Figure 3A), class *Bacilli* (Figure 3B), order *Bacillales* (Figure 3C), family *Bacillaceae* (Figure 3D) and species *B. subtilis* (Figure 3F) levels, with no consistent migratory shifts observed for these higher taxa. In contrast, genus-level profiling of *Bacillus* revealed marked intergroup variation (Figure 3E), its relative abundance was significantly higher in M1 before the migration than in M2 individuals after the migration. To explore shifts in interspecies microbial interactions driven by long-distance migration, genus-level circular co-occurrence networks were constructed for gut microbiota of *C. cygnus* sampled before and after the migration, with a consistent correlation threshold of |*r*| ≥ 0.85. Red edges denote significant positive correlations (*r* ≥ 0.85), whereas blue edges represent significant negative correlations (*r* ≤ −0.85). The network constructed from before the migration (Figure 3G) exhibited dense positive associations and almost no negative competitive links among bacterial genera, which indicated intense synergistic symbiosis and a tightly connected, stable gut microbial community before migration. In contrast, the network built from after the migration (Figure 3H) revealed moderately reduced overall connectivity and numerous newly formed negative correlation edges, proving that long-distance migration drastically reshaped the structural patterns of intestinal microbial interactions.

### 3.3 Isolation of probiotic *Bacillus* spp.

**Figure 4:**
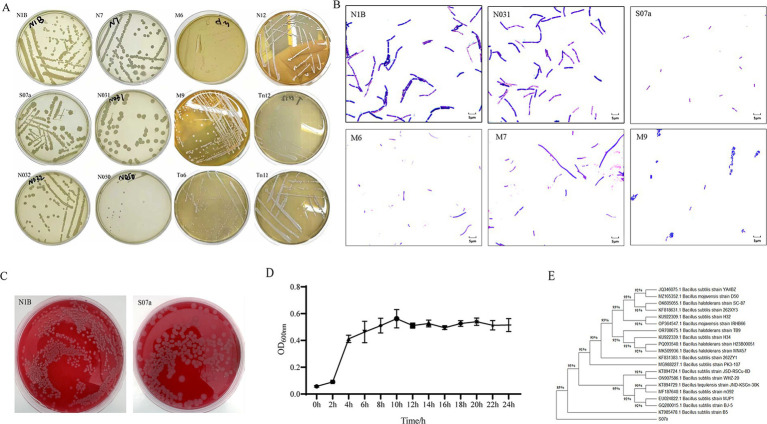
Preliminary screening and Characterization of probiotic *Bacillus* strains. **(A)** Colony morphology of 6 strains of *Bacillus*. **(B)** Gram staining results for some strains. **(C)** Hemolysis test of two strains of *Bacillus*. **(D)** Growth curve. **(E)** Evolutionary tree of the relationships between the isolated strains and *Bacillus subtilis.*

According to the purification of the morphology of the colonies, 12 strains of single colonies were obtained. The morphology of the colonies is shown in Figure 4A. A preliminary determination of the isolated strains of gram-positive rod bacteria is shown in Figure 4B. Hemolysis tests revealed that except for the N1B and S07a strains, the remaining strains had different degrees of hemolysis (Supplementary Figure S1). Further verification of the hemolysis of two nonhemolytic strains, *Bacillus* N1B and S07a, revealed that they had no hemolytic activity (Figure 4C), and further research on N1B and S07a was conducted.

### 3.4 Growth rate

To determine the growth characteristics of *B. subtilis* S07a, its OD value was determined, and a growth curve was constructed after 24 h of culture at 37 °C. After being cultivated for 2 h, *B. subtilis* S07a began to enter the logarithmic growth period, after which it entered the platform period; after 24 h, *B. subtilis* S07a was still in the plateau period, and the activity of the strain tended to stabilize (Figure 4D).

### 3.5 Acid and bile salt tolerance

To determine whether the S07a and N1B strains can survive under gastrointestinal conditions, the tolerance of the two strains to different pH values and different bile salts were studied. The survival rate of both strains of bacteria to pH 2–5 is approximately 50%, but the tolerance of S07a to acid seems to be greater than that of N1B. S07a showed extensive tolerance to bile salts (Table 3).

**Table 3:** Survival rate of the isolated strains (%).

Isolated	pH			Bile salt concentrations		
	2.0	3.0	5.0	0.3%	0.2%	0.1%
S07a	45.45 ± 2.75	51.93 ± 4.51	57.61 ± 5.32	55.82 ± 1.32	58.78 ± 4.05	66.27 ± 4.84
N1B	41.95 ± 3.86	48.81 ± 3.81	54.72 ± 2.04	45.75 ± 1.86	47.27 ± 3.75	52.90 ± 1.25

### 3.6 Antibiotic susceptibility and antibacterial test

Antibiotic sensitivity is a prerequisite for the screening of probiotics. Fifteen commonly used antibacterial drugs were selected for drug sensitivity tests on S07a and N1B. In addition to being moderately sensitive to erythromycin, strain S07a was sensitive to the remaining 14 antibacterial drugs; N1B was sensitive to the remaining 13 antibacterial drugs, except for its moderate sensitivity to clindamycin and rifampin (Supplementary Table S2). S07a has an inhibitory effect on *E. coli*, *Salmonella* and *S. aureus*, among which it has a strong inhibitory effect on *S. aureus*, but N1B has no inhibitory effect on all three strains of pathogenic bacteria (Table 4). S07a, which has good antibacterial effects, was selected for the next test.

**Table 4:** Bacteriostatic test results of isolated bacteria.

Tested bacteria	Isolated (mm)		
	Control	S07a	N1B
*Staphylococcus aureus*	6.0 ± 0.00	22.32 ± 0.19	6.2 ± 0.40
*Escherichia coli*	6.0 ± 0.00	16.87 ± 0.69	6.4 ± 0.34
*Salmonella typhi*	6.0 ± 0.00	10.09 ± 0.33	6.6 ± 0.7

The data are expressed as the mean ± SD. No bacteriostatic effect (diameter < 10 mm), moderate bacteriostatic effect (10 mm < diameter< 15 mm), or high bacteriostatic effect (diameter > 15 mm).

### 3.7 Biochemical identification and phylogenetic analysis of the S07a strain

Biochemical identification strips were used to determine the biochemical characteristics of the isolated strains, which were in line with the characteristics of *Bacillus* (Supplementary Table S3). The phylogenetic position was determined by 16S rRNA sequencing. Sequencing revealed that the S07a strain was highly homologous to the “KT985478.1 *B. subtilis* strain B5.” The sequencing results were compared and analysed by BLAST on NCBI, and the results were used to construct an evolutionary tree (Figure 4E). The sequence of the isolated strain was more than 99% similar to the genome sequence of *B. subtilis* published on NCBI. Therefore, the isolated strain was determined to be *B. subtilis* and was named *B. subtilis* S07a.

### 3.8 Acute toxicity animal study

**Figure 5:**
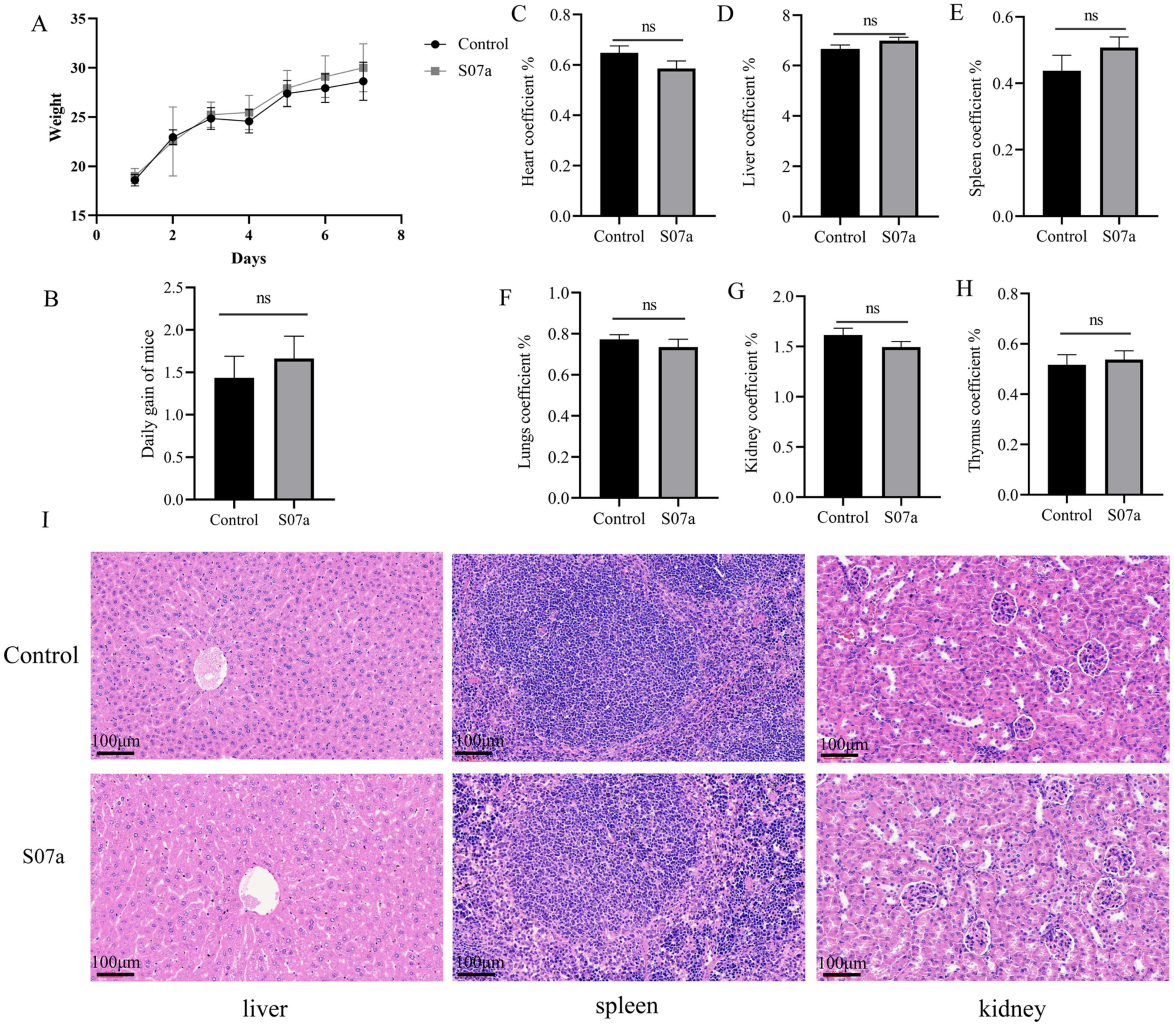
Results of the acute toxicity study (*n* = 14). **(A)** Changes in weight of the mice. **(B)** Daily gain of the mice. **(C–H)** Organ coefficients of the mice, including the heart, liver, spleen, lung, kidney, and thymus. **(I)** H&E-stained liver, spleen and kidney (*n* = 3). Magnification: ×20 for the liver, spleen and kidney. The data are shown as the mean ± SD; ns: *p* > 0.05, indicating that there was no significant difference between the control and S07a groups.

To study the safety of *B. subtilis* S07a, an acute toxicity test was used to irrigate the stomach with 1 × 10^9^ CFU/mL S07a in mice. After 7 days of observation, no clinical signs of abnormal physical activity changes, mental state or toxicity were detected in the mice. There was no significant difference in weight, daily weight gain (Figures 5A,B), organ index or blood cell indicators between the control group and the S07a group (*p* > 0.05) (Figures 5C–H) (Supplementary Table S4). The liver, spleen and kidney tissue sections were normal, and there were no pathological changes (Figure 5I). These findings indicate that the concentration of 1 × 10^9^ CFU/mL S07a is nontoxic to mice and may be beneficial to animal health.

### 3.9 Subacute toxicity animal study

**Figure 6:**
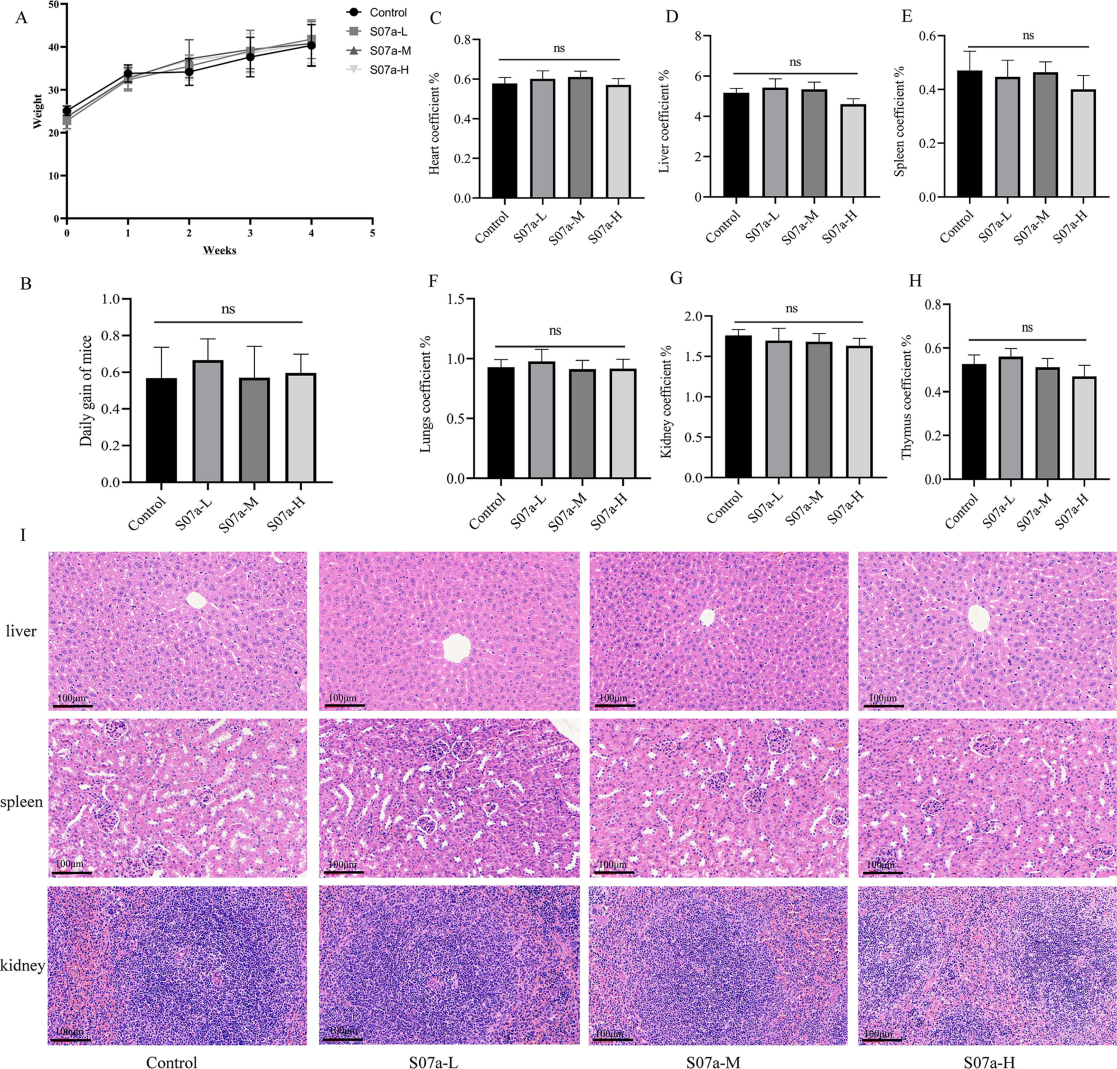
Results of the subacute toxicity study (*n* = 14). **(A)** Changes in Weight of the mice. **(B)** Daily gain of the mice. **(C–H)** Organ coefficients of the mice, including the heart, liver, spleen, lung, kidney, and thymus. **(I)** H&E-stained of liver, spleen and kidney (*n* = 3). Magnification: ×20 for the liver, spleen and kidney. The data are shown as the mean ± SD; ns: *p* > 0.05, indicating that there was no significant difference between the control and S07a groups.

In terms of subacute toxicity, the safety of the strain was further evaluated for 28 d. Compared with those in the control group, the weight gain rate, daily weight gain (Figures 6A,B), organ index (Figures 6C–H) and blood cell indicators (Supplementary Table S5) significantly increased in the S07a group. There was no significant difference (*p* > 0.05) in the liver, spleen and kidney tissue sections, which were normal, and there were no pathological changes (Figure 6I). Therefore, the safety evaluation of S07a *in vivo* is good.

### 3.10 Maintain of intestinal barrier function

**Figure 7:**
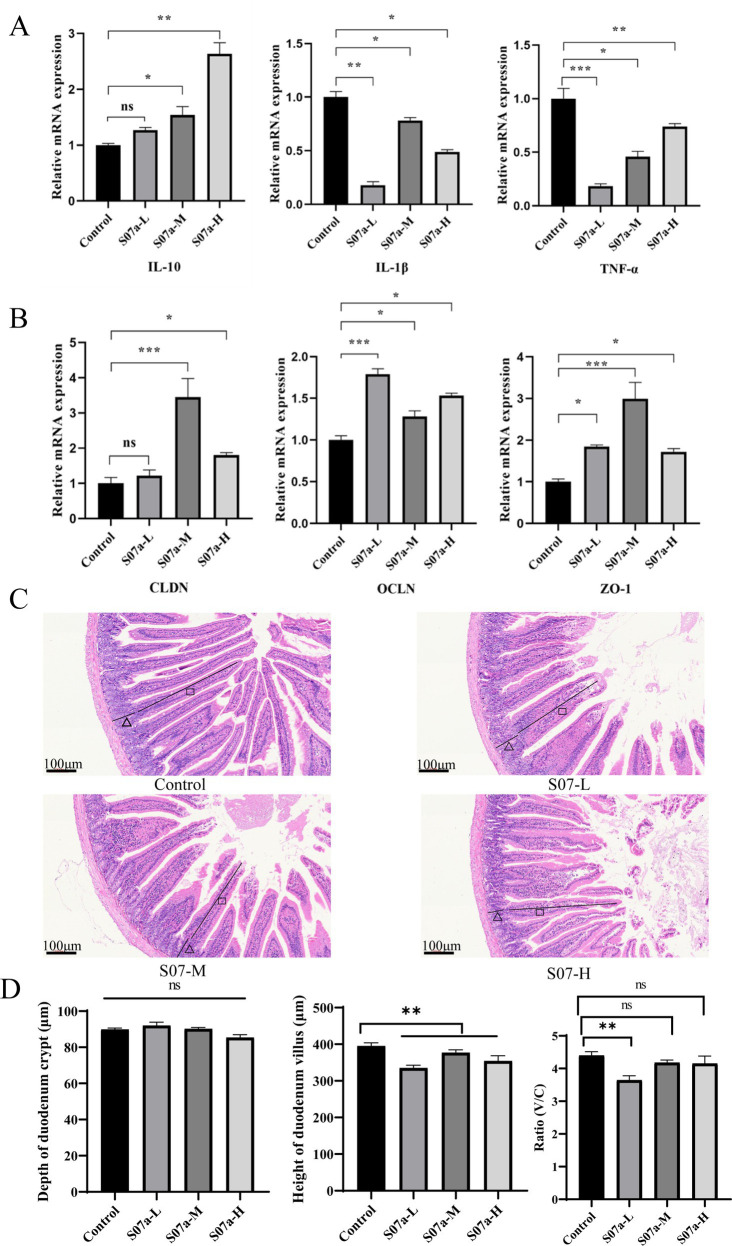
Effects of *Bacillus subtilis* S07a on the mRNA expression levels of tight junction protein **(A)** and inflammatory cytokines **(B)** in mice. H&E staining of the duodenum **(C)**. Statistical analysis of duodenal crypt depth, villus height and villus height/crypt depth (V/C) ratio in each group **(D)**. Histopathology revealed that the intestinal mucosa was structurally intact and that the intestinal villi were neatly arranged. H&E results of duodenum in each group at ×10 magnification. Data are shown as mean ± SD (*n* = 3); ns: *p* > 0.05 indicates that there was no significant difference between the control and S07a groups, *p < 0.05, **p < 0.01 indicates a significant difference between the control and S07a groups.

To determine whether *B. subtilis* S07a can maintain intestinal barrier function, the expression levels of tight junction related factors (*CLDN*, *OCLN*, *ZO-1*) and the level of interleukin (*IL-10*, *IL-1β*, *TNF-α*) were detected. Compared with that in the control group, the mRNA expression of the closely linked proteins *CLDN*, *OCLN* and *ZO-1* in the S07a group was significantly greater (*p* < 0.05) (Figure 7A); The mRNA expression of *IL-10* in the S07a group was significantly greater (*p* < 0.05); and the mRNA expression of *IL-1β* and *TNF-α* was significantly lower (*p* < 0.05) (Figure 7B). After gastric lavage S07a 28 d, the small intestine tissue of the mouse was normal and did not cause damage to the small intestine mucosa, and the length and density of the small intestinal villi were normal (Figure 7C). Histological examination of villus height and crypt depth of the duodenum revealed that no significant intergroup difference was observed in crypt depth among all four groups, and the villus height was significantly higher in the control than the S07-L, S07-M and S07-H group. The V/C values were significantly lower in the S07-L groups than in the control group and no significant difference was observed in the S07-M and S07-H (Figure 7D).

## 4 Discussion

In our research, we explored the changes in the structure and function of the gut microbiota before and after the migration of *C. cygnus* using metagenomic sequencing technology. Moreover, *B. subtilis* S07a from *C. cygnus* feces was isolated and screened to analyse its biological characteristics and evaluate its safety.

Our results clearly show that there are great differences in intestinal microbial communities before and after the migration of *C. cygnus*. This is largely because the formation of the intestinal microflora of *C. cygnus* is affected by various factors, such as migration, diet and the habitat environment (Risely et al., 2018; Momtaz et al., 2014). First, long-distance transregional migration in *C. cygnus* induces marked shifts in energy metabolism. Sustained flight raises the requirement for lipid catabolism, and selectively enriches fermentative taxa such as *Clostridium* to improve nutrient extraction efficiency (Trevelline et al., 2023). This aligns with our finding that the relative abundance of the genus *Clostridium* increased after the migration. Second, differences in food availability and water conditions among various overwintering habitats directly alter the gut community structure of *C. cygnus*. Before migration, *C. cygnus* rely predominantly on low-energy aquatic plants for nutrition; after migration, corn and other grains become their staple diet, consequently causing changes in the abundance of the *Firmicutes* phylum (Liu et al., 2022). Third, *C. cygnus* inhabit lakes, wetlands and farmlands throughout distinct migratory stages, and these habitats differ greatly in water quality and indigenous microbial communities. Exogenous microorganisms continuously colonize the intestinal tract and reshape gut microbiota composition (Zhang et al., 2024). In terms of the composition of the gut microbiota, *Firmicutes* and *Pseudomonadota* are the core phyla of *C. cygnus*, which is consistent with the findings of Wang et al. (2021). Notably, at the order level, the abundance of order Bacillales was greater in the Y1 group and the M1 group before migration. *Firmicutes* is an important metabolic phylum in the intestine that can decompose complex sugars, polysaccharides and fatty acids in food and produce energy and nutrients for animal absorption and utilization (Roggenbuck et al., 2015; Bennett et al., 2013). In the literature, bacteria enriched at the order level usually have rich metabolic pathways and strong environmental adaptability (Mi et al., 2023). At the genus and species level, the Y1 and Y2 groups were enriched in *Helicobacter pylori*. Momtaz et al. (2014b) and others reported that *H. pylori* is present in the stomachs of cattle, sheep and goats and is the natural host of *H. pylori*. Interestingly, wild animals often carry most zoonotic pathogens, and human activities are important factors affecting the level of pathogen carrying (Garcias-Bonet et al., 2024). Ecotourism activities may increase the likelihood of close contact between humans and wild animals, leading to an increase in the risk of transmission of zoonotic diseases between the two (Fackelmann et al., 2021). This kind of contact may also affect the community structure of the gut microbiota of wild animals, increasing the risk of disease transmission. After the migration of *C. cygnus* (in the Y2/M2 groups), the abundance of *Bacillota* (*Firmicutes* phylum) significantly decreased, whereas that of *Fusobacteriota* (*Fusobacteria* phylum) significantly increased in the M2 group. Members of *Bacillota* are widely known to participate in energy harvesting and metabolism, which are critical for fuelling long-distance flight (Waite and Taylor, 2014). The observed reduction in the abundance of *Bacillota* may reflect the high energy consumption and physiological stress caused by migration, whereas the increase in the abundance of *Fusobacteriota* may be associated with postmigratory recovery and environmental adaptation. These microbial shifts have clear ecological significance and are closely linked to energy metabolism during *C. cygnus* migration.

In addition, LEfSe analysis revealed that the relative abundance of *Clostridium* in the intestine of *C. cygnus* increased significantly after migration. *Clostridium* bacteria grow well in organic-rich and hypoxic environments such as the intestine, and can affect the host immune response by interacting with the host immune system. *Fusobacterium* can produce butyrate, which in turn promotes fat accumulation in the body and enhances immunity (Kint et al., 2022). The carbohydrate metabolism and energy metabolism functions of *C. cygnus* are reduced after migration, the source of food intake may be reduced during migration, and the energy demand is reduced. These results are directly related to the abundance of the *Firmicutes* phylum. *Cygnus cygnus* is a key species of wetland ecosystem. Its presence helps maintain the ecological balance of wetlands and is also an indicator of ecosystem health (Li et al., 2020). By studying the migration route, habitat selection and population dynamics of *C. cygnus*, we can better understand the functions and changes of wetland ecosystems (Wang et al., 2021). Every winter, large numbers of migratory birds gather, and intestinal pathogens can easily spread among birds, poultry, and livestock through feces. These findings suggest that while protecting *C. cygnus*, we should also pay attention to the prevention of zoonotic diseases (Binda et al., 2020).

We successfully isolated *B. subtilis* strains with favorable probiotic capacities from *C. cygnus*, while metagenomic data revealed obvious migratory fluctuations of the entire Bacillus genus rather than targeted enrichment of *B. subtilis*. This discrepancy indicated that the overall beneficial bacilli community underwent dramatic reshaping between pre-migration and post-migration stages, yet this shift was driven by multiple co-occurring *Bacillus* species rather than *B. subtilis* alone. Metagenomic abundance profiling detected markedly elevated loads of *Bacillus* spp. in *C. cygnus* before the migration; these microbes likely boosted carbohydrate energy extraction and sustained intestinal homeostasis to sustain strenuous long-distance flight (Trevelline et al., 2023). This observation confirms that native bacilli act as critical symbionts supporting the host adaptive capacity during migration (Wu et al., 2018). Meanwhile, our isolated *B. subtilis* serves as a promising native probiotic candidate for health intervention in migratory waterbirds. Consistent with the nonhaemolytic results previously reported by Golnari et al. (2024) for *B. subtilis*, our isolates *B. subtilis* N1B and S07a also showed no haemolytic activity. The antibiotic sensitivity of probiotics is an important screening indicator. The transmission of antibiotic resistance genes may occur through plasmid structure and bacterial gene mutations, leading to new antibiotic-resistant strains (Li et al., 2020). However, studies have shown that antibiotic-resistant probiotics are present in the intestine and help balance the gut microbiota after the use of antibiotics (Spears et al., 2021). *Bacillus subtilis* S07a and N1B were sensitive to 15 common antibiotics. *B. subtilis* S07a has an inhibitory effect on common diarrhoeal pathogens, including *E. coli*, *Salmonella* and *S. aureus*. The inhibitory effect on *S. aureus* is more obvious. *Bacillus subtilis* has been proven to have a good inhibitory effect on *S. aureus* (Zhang et al., 2025). In this study, 7-day acute toxicity and 28-day subacute toxicity tests in mice revealed no abnormal mental state or normal weight gain after intragastric administration of *B. subtilis* S07a, which was the same as the safety test effect of a strain of the *Lactobacillus* probiotic JJB3(Halder et al., 2017). Our biosafety assessment only relied on hematological data to exclude systemic infection. Limited sample volume and experimental timeline precluded us from performing fecal shedding and bacterial translocation assays, restricting the comprehensiveness of our risk evaluation. We plan to incorporate these bacteriological measurements into subsequent long-term animal trials to build a more complete safety profile. Excessive mRNA expression of *IL-1β* and *TNF-α* leads to a significant increase in the permeability of the intestinal epithelium. *Clostridium* can promote the secretion of *IL-10* (Haque et al., 2024). In addition to direct immune activation, *IL-1β* promotes intestinal inflammation by destroying the close connection barrier of the intestine and increasing the intestinal permeability of the lumen antigen (Rawat et al., 2020). Probiotics can improve the health of the host by increasing intestinal barrier function and regulating the intestinal microbiota (Chandrasekaran et al., 2024). This study revealed that *B. subtilis* S07a significantly increased the expression of *CLDN*, *OCLN*, and *ZO-1*, which was consistent with the findings of Zhou et al. (2022). Thus, *B. subtilis* S07a reduces anti-inflammatory effects and enhances intestinal health.

## 5 Conclusion

In short, the macrogenome results in a decrease in the abundance of microorganisms related to carbohydrate decomposition in the intestines of the *C. cygnus* after migration. The isolated *B. subtilis* S07 has good *in vitro* probiotic potential and high *in vivo* safety. In addition, *B. subtilis* S07a has the potential to be anti-inflammatory and enhance intestinal barrier function. It can be developed and applied as a candidate strain of probiotic preparations from the *C. cygnus*.

## Data Availability

The raw metagenomic sequencing data generated in this study have been deposited in the NCBI Sequence Read Archive (SRA) under BioProject accession number PRJNA1489321 (https://www.ncbi.nlm.nih.gov/sra/PRJNA1489321).
