# Supplementary material for: Metagenomic analysis of the gut microbiota in Cygnus cygnus and isolation, identification, and safety assessment of Bacillus
Source: Front Microbiol. 2026 Jul 16;17:1898323. doi: 10.3389/fmicb.2026.1898323 (PMC13422541; doi:10.3389/fmicb.2026.1898323)
Supplement: Supplementary file 1 [file Supplementary_file_1.DOCX]

**Supplementary Materials**


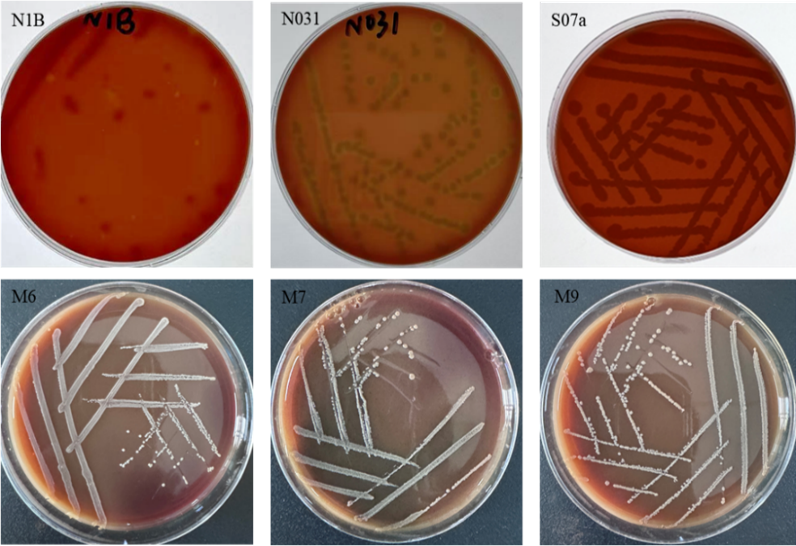


**Fig. S1** Hemolysis test results for some strains

**Table S1** Data information

| Sampling position | Sampling time | Sample name | Group | Clean_GC (%) | Effective (%) |
| --- | --- | --- | --- | --- | --- |
| Yili Cygnus cygnus Spring Wetland Park | March 2023 | Y01 | Y1 | 41.92 | 96.83 |
| Yili Cygnus cygnus Spring Wetland Park | March 2023 | Y02 | Y1 | 46.69 | 99.35 |
| Yili Cygnus cygnus Spring Wetland Park | March 2023 | Y03 | Y1 | 42.02 | 98.03 |
| Yili Cygnus cygnus Spring Wetland Park | March 2023 | Y04 | Y1 | 52.5 | 98.62 |
| Yili Cygnus cygnus Spring Wetland Park | March 2023 | Y05 | Y1 | 39.79 | 97.45 |
| Yili Cygnus cygnus Spring Wetland Park | March 2023 | Y06 | Y1 | 41.88 | 98.1 |
| Yili Cygnus cygnus Spring Wetland Park | December 2023 | E01 | Y2 | 56.39 | 98.3 |
| Yili Cygnus cygnus Spring Wetland Park | December 2023 | E02 | Y2 | 52.18 | 97.63 |
| Yili Cygnus cygnus Spring Wetland Park | December 2023 | E03 | Y2 | 46.91 | 98.04 |
| Yili Cygnus cygnus Spring Wetland Park | December 2023 | E04 | Y2 | 54.79 | 98.87 |
| Yili Cygnus cygnus Spring Wetland Park | December 2023 | E05 | Y2 | 40.59 | 97.85 |
| Yili Cygnus cygnus Spring Wetland Park | December 2023 | E06 | Y2 | 49.65 | 95.64 |
| Manas National Wetland park | March 2023 | M01 | M1 | 31.45 | 99.08 |
| Manas National Wetland park | March 2023 | M02 | M1 | 46.45 | 99.46 |
| Manas National Wetland park | March 2023 | M03 | M1 | 35.08 | 97.63 |
| Manas National Wetland park | March 2023 | M04 | M1 | 40.5 | 94.37 |
| Manas National Wetland park | March 2023 | M05 | M1 | 32.81 | 98.78 |
| Manas National Wetland park | March 2023 | M06 | M1 | 32.76 | 98.69 |
| Manas National Wetland park | December 2023 | N01 | M2 | 47.83 | 96.71 |
| Manas National Wetland park | December 2023 | N02 | M2 | 36.26 | 98.37 |
| Manas National Wetland park | December 2023 | N03 | M2 | 40.83 | 97.56 |
| Manas National Wetland park | December 2023 | N04 | M2 | 40.55 | 97.61 |
| Manas National Wetland park | December 2023 | N05 | M2 | 36.65 | 98.47 |
| Manas National Wetland park | December 2023 | N06 | M2 | 36.47 | 98.48 |

**Table S2** Results of the drug sensitivity test

| Types of antibiotics | Antibiotics name | Drug concentration | Judgement standard:antibacterial ring diameter/mm | | | Susceptibility | |
| --- | --- | --- | --- | --- | --- | --- | --- |
|  |  |  | Resistance(R) | Intermediate(I) | Sensitive(S) | S07a | N1B |
| β-Lactams | Penicillin G | 10 U | ≤19 | 20 ~ 27 | ≥28 | S | S |
|  | Ampicillin | 10 μg | ≤18 | 19 ~ 25 | ≥26 | S | S |
|  | Imipenem | 10 μg | ≤13 | 14 ~ 15 | ≥16 | S | S |
| Aminoglycosides | Amikacin | 30 μg | ≤14 | 15 ~ 16 | ≥17 | S | S |
|  | Gentamicin | 10 μg | ≤12 | 13 ~ 14 | ≥15 | S | S |
|  | Kanamycin | 30 μg | ≤13 | 14 ~ 17 | ≥18 | S | S |
| Quinolones | Ciprofloxacin | 5 μg | ≤15 | 16 ~ 20 | ≥21 | S | S |
|  | Levofloxacin | 5 μg | ≤14 | 15 ~ 17 | ≥18 | S | S |
| Tetracycline | Tetracycline | 30 μg | ≤14 | 15 ~ 18 | ≥19 | S | S |
| Lincomycins | Clindamycin | 2 μg | ≤14 | 15 ~ 20 | ≥21 | S | I |
| Chloramphenicol | Chloramphenicol | 30 μg | ≤12 | 13 ~ 17 | ≥18 | S | S |
| Peptides | Vancomycin | 30 μg | ≤8 | 9 ~ 12 | ≥13 | S | S |
| Macrolides | Erythromycin | 15 μg | ≤13 | 14 ~ 22 | ≥23 | I | S |
| Sulfonamides | Compound Sulfamethoxazole | 25 μg | ≤10 | 11 ~ 15 | ≥16 | S | S |
| Rifampin | Rifampin | 5 μg | ≤16 | 17 ~ 19 | ≥20 | S | I |

The results were judged with reference to “Standards for Antibacterial Drug Susceptibility Test by Disk Method” WS/T 125–1999. S sensitive, I intermediate, R resistant

**Table S3** Biochemical identification results for S07a

| Biochemical index | S07a |
| --- | --- |
| Anaerobic growth | + |
| Citrate | + |
| V-P test | + |
| Propionate | + |
| D-xylose | ﹣ |
| L-Arabinose | + |
| D-Mannitol | ﹣ |
| Gelatin liquefaction | + |
| 7% Sodium chloride | + |
| pH 5.7 growth | + |
| Nitrate reduction | + |
| Starch hydrolysis | ﹣ |

“ + ” denotes a positive reaction “ - ” denotes a negative reaction. Refer to Bergey’s Bacterial Identification Manual (Eighth Edition) for the determination of results.

**Table S4** Effects of *Bacillus subtilis* S07a on blood cell indices in mice after gavage

| Items | Control | S07a |
| --- | --- | --- |
| WBC（10^9^/L） | 4.79 ± 1.68 | 4.24 ± 1.47 |
| LYM（10^9^/L） | 3.23 ± 1.33 | 2.55 ± 0.95 |
| MON（10^9^/L） | 0.55 ± 0.24 | 0.97 ± 0.62 |
| GRA（10^9^/L） | 1.01 ± 0.42 | 0.72 ± 0.28 |
| RBC（10^12^/L） | 8.54 ± 1.10 | 8.31 ± 1.40 |
| HGB（g/L） | 149.19 ± 21.24 | 140.14 ± 28.89 |

**Table S5** Effects of *Bacillus subtilis* S07a on blood cell indices in mice after gavage

| Items |  | | | |
| --- | --- | --- | --- | --- |
|  | Control | S07a-L | S07a-M | S07a-H |
| WBC（10^9^/L） | 5.39 ± 2.77 | 6.19 ± 2.31 | 5.70 ± 1.49 | 7.04 ± 1.50 |
| LYM（10^9^/L） | 2.89 ± 1.81 | 3.04 ± 1.26 | 3.07 ± 1.05 | 3.81 ± 1.25 |
| MON（10^9^/L） | 1.75 ± 1.01 | 2.28 ± 1.49 | 1.74 ± 0.68 | 2.27 ± 0.94 |
| GRA（10^9^/L） | 0.75 ± 0.26 | 0.87 ± 0.30 | 0.89 ± 0.26 | 0.95 ± 0.24 |
| RBC（10^12^/L） | 10.90 ± 1.28 | 10.03 ± 1.95 | 10.33 ± 2.04 | 11.13 ± 0.87 |
| HGB（g/L） | 177.51 ± 25.92^a^ | 164.54 ± 35.41^b^ | 171.28 ± 36.97^a^ | 189.16 ± 18.0^b^ |
